# Volvulus du mesentere sur lipome mesenterique: à propos d’un cas

**DOI:** 10.11604/pamj.2016.25.55.10382

**Published:** 2016-09-30

**Authors:** Hamidou Dème, Nfally Badji, Léra Géraud Akpo, Mouhamed Hamine Touré, Ronald Draha, Fallou Gallas Niang, Abdoulaye Dione Diop, El Hadj Niang

**Affiliations:** 1 Service de Radiologie et Imagerie Médicale, CHU Aristide Le Dantec, BP 3001 Pasteur Dakar, Sénégal; 2 Service de Radiologie et Imagerie Médicale, CHNU Fann, Avenue Cheikh Anta Diop Dakar, Sénégal

**Keywords:** Lipome mésentérique, volvulus, échographie, TDM, Mesenteric lipoma, volvulus, ultrasound, CT scan

## Abstract

Nous rapportons le cas d'une patiente de 7 ans qui a consulté pour une douleur abdominale aigue paroxystique siégeant au niveau de l'épigastre associée à des vomissements sans arrêt des matières et des gaz. L'examen clinique ainsi que le bilan biologique étaient sans particularité. L'échographie abdominale demandée en première intention a montré une masse du flanc et de la fosse iliaque droite échogène, homogène à contours réguliers sans signal vasculaire au Doppler associée à un signe du tourbillon des vaisseaux mésentériques. A la tomodensitométrie cette masse correspondait à une formation lipomateuse, bien limitée, exerçant un effet de masse sur le cœcum avec volvulus du mésentère au contact. La disposition des vaisseaux mésentériques à leur origine était normale. Le diagnostic de volvulus du mésentère sur lipome a été retenu. La prise en charge chirurgicale et l'analyse anatomopathologique de la pièce opératoire a confirmé le diagnostic. Nous allons à travers ce cas clinique revoir l'apport de l'échographie et du scanner dans le diagnostic de volvulus du mésentère.

## Abstract (en)

We report the case of a 7-year old patient presenting for acute paroxysmal abdominal pain at the level of epigastrium associated with vomiting without involving blockage of materials and gas. Clinical examination and laboratory tests were unremarkable. Abdominal ultrasound was requested as a first line investigationa and showed a right echogenic homogeneous flank and iliac fossa mass with regular contours without vascular Doppler signal and associated with “whirl sign“ of mesenteric vessels. On CT scan this mass was seen as a well-defined lipoma formation, exerting a mass effect on the cecum, which was in direct contact with mesenteric volvulus. Arrangement of the mesenteric vessels at their origin was normal. The diagnosis of mesenteric volvulus associated with lipoma was retained. Surgical management and histological analysis of the surgical specimen confirmed the diagnosis. This clinical case aims to highlight the contribution of ultrasound and CT scan in the diagnosis of midgut volvulus.

## Introduction

Le lipome est une tumeur bénigne du tissu adipeux. Habituellement localisé au niveau des membres et du tronc, sa localisation au niveau du mésentère est rare [1, 2]. Peu décrit chez l'enfant dans la littérature, le lipome du mésentère est une cause secondaire du volvulus du grêle [1–4]. Nous rapportons un cas de volvulus du mésentère sur lipome chez une patiente de 7 ans chez qui l'échographie et la tomodensitométrie nous ont permis de poser le diagnostic.

## Patient et observation

**Figure 1:**
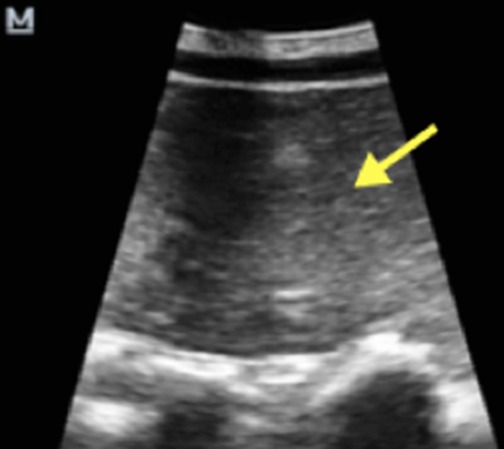
Coupe longitudinale d’échographie abdominale avec une sonde convexe montrant une masse échogène avec des stries linéaires hyperéchogènes (flèche jaune) au niveau du flanc et de la fosse iliaque droite

**Figure 2:**
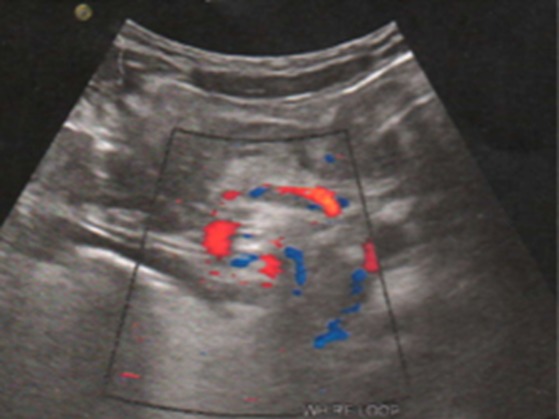
Coupe transversale d’échographie abdominale avec sonde convexe en mode B couplé au doppler montrant une image de tourbillon (« whirl sign ») autour des vaisseaux mésentériques en rapport avec un volvulus

**Figure 3:**
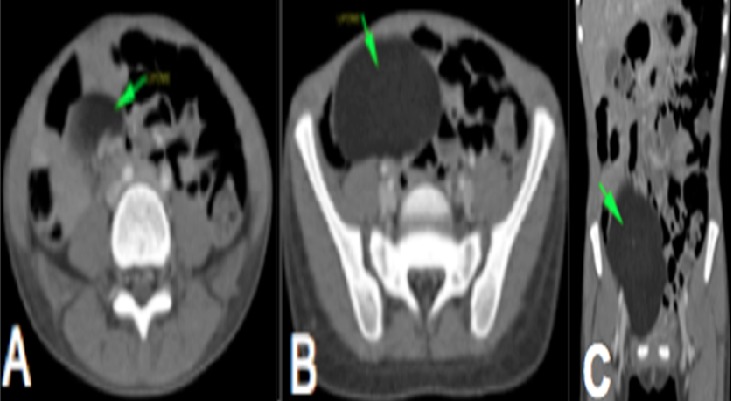
A et B): Coupes axiales de scanner abdominal avec injection de produit de contraste montrant une masse de densité graisseuse homogène non rehaussée au niveau du flanc et de la fosse iliaque droite comprimant le cœcum en rapport avec un lipome C): reconstruction coronale de scanner abdominal injecté montrant la masse graisseuse et ses limites

**Figure 4:**
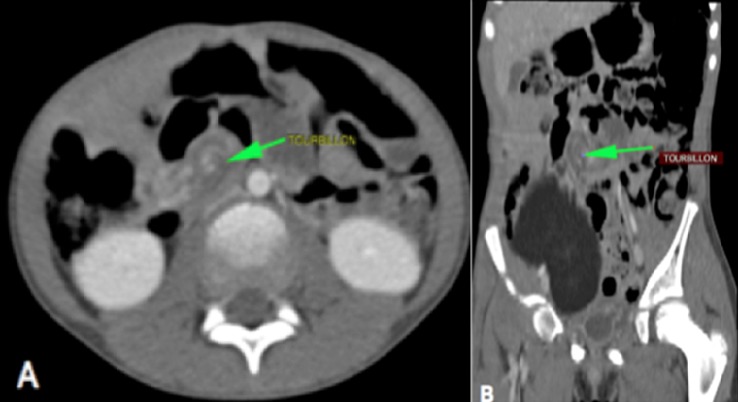
A) Coupe axiale; B) reconstruction coronale de scanner abdominal injecté montrant le sign du tourbillon (flèche verte) au niveau du mésentère juste au dessus de la masse lipomateuse en rapport avec le volvulus du mésentère

**Figure 5:**
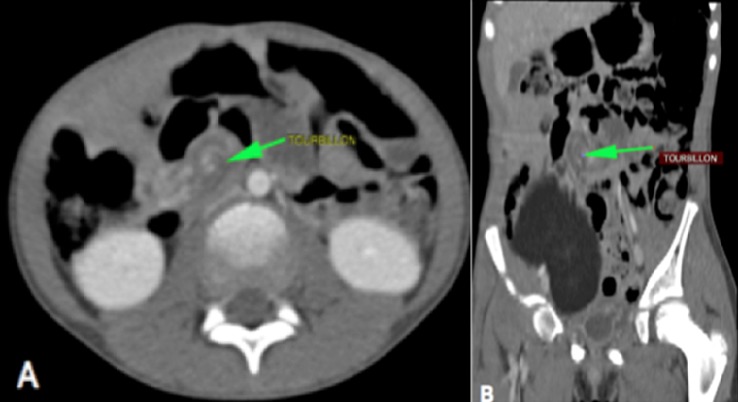
Coupe axiale de scanner abdominal injecté montrant l’artère mésentérique supérieure (flèche rouge) et la veine mésentérique supérieure (flèche bleue) de disposition normale en dessous du confluent spléno-mésaraïque

Il s'agissait d'une fille de 7 ans, reçue pour douleur abdominale aigue paroxystique évoluant depuis 4 jours de siège épigastrique associée à des vomissements sans trouble du transit. On notait un long passé (4 ans) de douleurs abdominales intermittentes, spontanément résolutives. A l'examen clinique, les constantes étaient normales, l'abdomen était plat, il n'y avait pas de défense ou de contracture. On ne palpait pas de masse ou d'organomégalie. Les examens biologiques réalisés étaient sans anomalies. L'échographie abdominale demandée en première intention, réalisée avec une machine de marque Mindray DC7 à l'aide d'une sonde convexe de basse fréquence (3,5 à 5MHz) avait trouvé une formation tissulaire intra-abdominale au niveau du flanc droit et de la fosse iliaque droite, échogène avec des stries linéaires hyperéchogènes de taille difficile à préciser, de contours réguliers (Figure 1). Elle exerçait un effet de masse sur les anses avoisinantes avec un ralentissement du péristaltisme intestinal, une stase gastrique et duodénale. Au Doppler, on notait un caractère avasculaire de la masse. Cependant, il existait un « whirl sign » au niveau des vaisseaux mésentériques (Figure 2). Les vaisseaux mésentériques supérieurs étaient de disposition normale à leur origine. Le foie, la rate, le pancréas et les reins étaient d'aspect normal. La tomodensitométrie réalisée en deuxième intention avec un scanner de marque Siemens Somatom Definition 64 barrettes, en contraste spontané puis après injection de produit de contraste au temps portal objectivait une masse homogène, de densité graisseuse (-93 UH), siégeant au niveau du flanc droit et de la fosse iliaque droite, non rehaussée après injection de produit de contraste (Figure 3). Elle mesurait 66 x 37mm de diamètres pour 115mm de hauteur et ne présentait pas de cloison ni de calcification. Elle comprimait et refoulait le cœcum. Au contact du pole supérieur de la masse, on notait un enroulement des anses, des vaisseaux mésentériques et de la graisse mésentérique donnant le classique signe du tourbillon ou « whirl sign » (Figure 4) en rapport avec un volvulus du mésentère. A leur origine, les vaisseaux mésentériques supérieurs avaient une disposition normale (Figure 5). Il n'y avait pas de distension des anses ou d'anomalie de rehaussement de leur paroi. Le diagnostic de volvulus du mésentère sur lipome a été retenu. La laparotomie exploratrice a mis en évidence une masse lipomateuse, qui a été réséquée suivie de la détorsion du mésentère. Il n'y avait pas de signe de souffrance des anses. Les suites opératoires étaient simples. L'analyse anatomopathologique de la pièce opératoire avait conclu à un lipome conventionnel.

## Discussion

Le lipome du mésentère ou du tractus gastro-intestinal est rare [2, 4]. Il est le plus souvent asymptomatique dans ses formes non compliquées [2–4]. Localisé préférentiellement au niveau de la racine du mésentère chez l'enfant ; il peut rester longtemps asymptomatique. Il est habituellement diagnostiqué quand sa taille augmente, devenant alors symptomatique, pouvant être révélé par des signes digestifs tels que les douleurs abdominales intermittentes, une anorexie, des vomissements, mais aussi par l'effet de masse qu'il peut exercer sur les anses digestives avoisinantes [1, 2, 5]. En effet chez notre patiente, on notait un tableau de douleur abdominale aigue associée à des vomissements sur un long passé de douleurs abdominales intermittentes. Le lipome peut être à l'origine d'une occlusion intestinale par volvulus du mésentère. Il représente environ 5% des causes de volvulus du grêle tout âge confondu [5]. Le volvulus du mésentère sur lipome a été rarement décrit chez l'enfant comme dans notre cas. Cliniquement suspecté, le diagnostic de volvulus du mésentère repose sur les examens d'imagerie qui permettent en plus du diagnostic positif de rechercher une étiologie. Les modalités d'imagerie que sont l'échographie et la tomodensitométrie permettant le diagnostic du lipome du mésentère et de ses complications [1–5]. L'échographie est l'examen de première intention devant des signes d'occlusion intestinale chez l'enfant [6]. Couplée au mode Doppler, l'échographie mode B est performante dans le diagnostic de volvulus du mésentère en montrant le classique « whirl sign » qui correspond à une masse constituée de graisse mésentérique et d'anses, présentant des tours de spires, correspondant à des anses grêles volvulées dont les parois sont vraisemblablement épaissies. Au Doppler couleur, les structures vasculaires sont repérées au centre de ce volvulus avec l'artère située à droite de la veine. On retrouve des signaux Doppler correspondant aux vaisseaux mésentériques supérieurs au sein des anses digestives volvulées, comme s'enroulant avec elles [7]. Cependant dans ce contexte, il faut éliminer un mésentère commun qui résulte d'une anomalie de rotation du tube digestif dont le diagnostic repose sur la mise en évidence à l'échographie d'une transposition des vaisseaux mésentériques supérieures avec la veine mésentérique supérieure qui est située à gauche de la l'artère mésentérique supérieure [6, 7]. Chez notre patiente l'échographie a permis d'évoquer le diagnostic de volvulus du mésentère en montrant le signe du tourbillon et a permis d'éliminer en même temps une malrotation intestinale en montrant une disposition normale des vaisseaux mésentériques supérieurs à leur origine.

Chez notre patiente l'échographie avait montré une masse échogène avec des stries linéaires hyperéchogènes faisant évoquer un lipome au contact du volvulus mésentérique. Dans la recherche étiologique du volvulus, l'échographie permet d'évoquer le lipome mésentérique en montrant une masse échogène au contact des anses [8]. Cependant le lipome profond peu avoir l'aspect d'une masse hypo, iso ou hyperéchogène, rendant le diagnostic moins évident [9] d'ou l'intérêt de la tomodensitométrie. La tomodensitométrie constitue la modalité d'imagerie de choix car ne confirme pas seulement la nature graisseuse homogène de la masse mais donne aussi des informations concernant l'effet sur les anses grêles, en montrant les signes de volvulus avec le classique « whirl sign » et en permettant d'apprécier la paroi des anses à la recherche de signes d'ischémie [2]. Dans notre cas, la tomodensitométrie avait montré des signes de volvulus du mésentère. Cependant il n'était pas noté de dilatation des anses ou de signe d'ischémie pariétale. A l'exploration chirurgicale il n'y avait pas de signe de souffrance intestinale et une détorsion simple a été réalisée avec résection de la masse. Comparée à l'échographie, la tomodensitométrie fournit des renseignements supplémentaires concernant la nature de la masse et permet de réaliser un éventuel bilan d'extension en cas de lésion maligne ce qui est indispensable avant toute intervention [1–4] Dans notre cas, le diagnostic de lipome a été retenu devant la densité graisseuse de la masse (-93 UH), l'aspect homogène, la paroi fine, l'absence de calcification et de cloisons et l'absence de rehaussement après injection de produit de contraste. Devant cette masse mésentérique d'autres diagnostics quoique peu probables pouvaient être évoqués notamment le liposarcome, le lipoblastome, le lymphangiome kystique mais leurs aspects tomodensitométriques sont différents de ceux du lipome [1, 4, 10, 11].

## Conclusion

Le volvulus du mésentère secondaire à un lipome est une affection rare. Son diagnostic repose sur l'imagerie. L'échographie est performante pour le diagnostic de volvulus du mésentère en montrant le classique « whirl sign » mais aussi permet d'évoquer le lipome du mésentère comme cause du volvulus. Cependant la tomodensitométrie en plus du diagnostic positif, permet d'avoir des détails anatomiques, une meilleure orientation étiologique en éliminant les diagnostics alternatifs.
